# Tumor volume change at radiation boost planning to estimate the response to chemoradiotherapy in stage III unresectable NSCLC (TORCH): a multicenter retrospective observational study

**DOI:** 10.1007/s00066-025-02374-3

**Published:** 2025-03-03

**Authors:** Simon Trommer, Jörg Andreas Müller, Michael Oertel, Felix Ehret, Siyer Roohani, Hai Minh Ha, Quynh Ngo Ha, Kathrin Hering, Franziska Nägler, Tim Lange, Matthias Mäurer, Thomas Weissmann, Florian Putz, Maike Trommer, Christian Baues, Sophie Dobiasch, Maria Waltenberger, Tomas Skripcak, Dirk Vordermark, Daniel Medenwald

**Affiliations:** 1 Department of Radiation Oncology, University Hospital Halle (Saale), Ernst-Grube-Str. 40, 06120 Halle (Saale), Germany; 2 Clinic for Radiotherapy—Radiooncology, University Hospital Münster, Münster, Germany; 3 Clinic for Radiooncology and Radiation Therapy, Charité—Universitätsmedizin Berlin, Berlin, Germany; 4 German Cancer Consortium (DKTK), partner site Berlin, a partnership between DKFZ and Charité—Universitätsmedizin Berlin, Berlin, Germany; 5 BIH Biomedical Innovation Academy, BIH Charité (Junior) Clinician Scientist Program, Berlin Institute of Health, Charité—Universitätsmedizin Berlin, Berlin, Germany; 6 University Clinic for Radiation Therapy, University Hospital Magdeburg A. ö. R, Magdeburg, Germany; 7 Department of Radiation Oncology, University of Leipzig, Leipzig, Germany; 8 Comprehensive Cancer Center Central Germany (CCCG), University of Leipzig Medical Center, Leipzig, Germany; 9 Department of Radiotherapy, Hannover Medical School, Germany; 10 Department for Radiotherapy and Radiation Oncology, University Hospital Jena, Friedrich-Schiller-University, Am Klinikum 1, 07747 Jena, Germany; 11 Clinician Scientist Program OrganAge, Interdisciplinary Center for Clinical Research (IZKF), Jena University Hospital, 07747 Jena, Germany; 12 Department of Radiation Oncology, Universitätsklinikum Erlangen, Friedrich-Alexander-Universität Erlangen-Nürnberg, Erlangen, Germany; 13 Department of Radiation Oncology, Faculty of Medicine and University Hospital Cologne, University of Cologne, Kerpener Str, 62, 50937 Cologne, Germany; 14 Department of Radiation Oncology, Olivia Newton-John Cancer Wellness & Research Centre, University of Melbourne, Austin Health, 145 Studley Rd, CIV 3084 Heidelberg, Australia; 15 Department of Radiooncology, Marien Hospital Herne, University Hospital, Ruhr-University Bochum, Herne, Germany; 16 Department of Radiation Oncology and Radiotherapy at the Klinikum rechts der Isar, Technical University of Munich, Munich, Germany; 17 Institute of Radiation Medicine (IRM), Helmholtz Zentrum München, Neuherberg, Germany; 18 German Cancer Consortium (DKTK), partner site Munich, a partnership between DKFZ and Klinikum rechts der Isar, Technical University of Munich, Munich, Germany German Cancer Consortium (DKTK) Dresden and German Cancer Research Center (DKFZ) Heidelberg, Munich, Germany; 19 German Cancer Consortium (DKTK), Partner Site Dresden, and German Cancer Research Center (DKFZ), Heidelberg, Dresden, Germany; 20 Department of Radiotherapy and Radiation Oncology, Faculty of Medicine and University Hospital Carl Gustav Carus, TUD Dresden University of Technology, Dresden, Germany

**Keywords:** Non-small cell lung cancer, Gross tumor volume, Immunotherapy, Durvalumab, Stage III lung cancer

## Abstract

**Background:**

Progression-free (PFS) and overall survival (OS) in UICC stage III non-small cell lung cancer (NSCLC) after definitive concurrent chemoradiotherapy (CRT) can be increased with consolidating immunotherapy. Recent studies have shown a strong predictive value of gross tumor volume (GTV) changes during CRT on OS. The TORCH trial investigated the prognostic impact of GTV changes during CRT as a predictor for a response to immunotherapy.

**Methods:**

This retrospective non-interventional observational multicenter trial included *n* = 203 patients from 10 German university centers for radiation oncology with confirmed inoperable NSCLC in UICC stage III A–C. Patients had received CRT between 2015 and 2023 as a curative-intent treatment approach. Patient and tumor characteristics were collected anonymously via electronic case report forms. Initial GTVs before CRT (initial planning CT, GTV1) and at 40–50 Gy (re-planning CT for radiation boost, GTV2) were delineated. Absolute and relative GTV changes before/during CRT were correlated with OS to predict the response to CRT with sequential immunotherapy. Hazard ratios (HR) of survival analyses were estimated using adjusted Cox regression models.

**Results:**

The mean GTV1 before radiation therapy (RT) was 145.29 ml with the 25th, 50th, and 75th percentiles being 61.36 ml, 145.29 ml, and 204.93 ml, respectively. Before initiation of the radiation boost, the mean GTV2 was 99.58 ml, with the 25th, 50th, and 75th percentiles at 32.93 ml, 70.45 ml, and 126.85 ml. The HR for the impact of GTV1 on survival was 0.99 per ml (95% confidence interval [CI] 0.99–1.00; *p* = 0.49). For the absolute volume change between GTV1 and GTV2, the HR was 1.004 per ml (95% CI 0.997–1.011; *p* = 0.26). In a subgroup analysis of patients who were treated with durvalumab, absolute volume changes between GTV1 and GTV2 were associated with longer OS (HR = 0.955 per ml; 95% CI 0.916–0.996; *p* = 0.03). Overall, durvalumab treatment was positively associated with OS, demonstrating an HR of 0.454 (95% CI 0.209–0.990; *p* = 0.047).

**Conclusion:**

Pretreatment GTV and absolute GTV volume changes did not significantly correlate with OS. However, the absolute volume change between the pretreatment and replanning GTV was associated with longer OS in patients treated with durvalumab. Histological subtype, grading, UICC stage, age at onset, pulmonary comorbidities, and smoking status had no significant association with OS. Durvalumab treatment was associated with improved OS.

**Supplementary Information:**

The online version of this article (10.1007/s00066-025-02374-3) contains supplementary material, which is available to authorized users.

## Introduction

Locally advanced non-small cell lung cancer (NSCLC) UICC stage III accounts for approximately one third of all NSCLC patients [1]. Surgical resection for operable and definitive concurrent chemoradiotherapy (CRT) for inoperable patients defines the standard of care in the interdisciplinary treatment of stage III NSCLC [2]. However, treatment outcomes after CRT are still suboptimal, with high local recurrence and poor median progression-free survival (PFS) rates [3, 4]. Thus, prognostic and predictive factors before and during CRT are needed to select the best therapeutic approach regarding patients’ survival probability. Well-selected elderly patients could benefit from CRT treatment followed by durvalumab, with comparable survival to younger patients [5]. Sequential CRT or radiotherapy (RT) alone is appropriate for frail patients unable to tolerate concurrent therapy [6].

The prognostic value of the gross tumor volume (GTV) in CRT of stage III NSCLC is under debate.

Despite the correlation between increasing tumor volume and higher T stages in the TNM system, stage was found to be a poor predictor of primary tumor volume [7, 8]. Further evidence suggests that the GTV is an important prognostic factor for NSCLC and small cell lung cancer (SCLC) patients, albeit not superior to the T stage [9, 10]. Guckenberger et al. investigated the role of adaptive RT in locally advanced NSCLC. In their findings, adaptation of RT to the shrinking GTV did not compromise dose coverage of volumes of suspected microscopic disease [11].

[^18^F]-2-Fluoro-2-deoxy-D-glucose (FDG) positron-emission tomography/computed tomography (PET/CT) plays an important role in treatment planning [12]. According to the National Comprehensive Cancer Network (NCCN) guidelines, FDG-PET/CT RT planning significantly improves targeting accuracy, especially for patients with significant atelectasis and when intravenous CT contrast is contraindicated [13]. A randomized trial of conventional PET- and CT-based irradiation plus elective nodal irradiation (conventional target group) versus PET-based RT planning demonstrated non-inferiority of the PET-based approach, with decreased recurrence rates and a trend towards similar toxicity rates [12].

The “young DEGRO” working group of the German Society of Radiation Oncology (DEGRO) conducted a multicenter study to evaluate the impact of PET/CT-based treatment planning on the prognosis of patients with NSCLC stage III. The use of PET/CT co-registration in radiation planning tended to result in better oncologic outcomes, although no significant association could be shown [14].

In the phase III randomized NCT02125461 trial (PACFIC), consolidating immunotherapy with the PD-L1 inhibitor durvalumab after radical concurrent chemoradiotherapy for stage III NSCLC demonstrated superior OS and progression-free survival (PFS) compared to placebo. Furthermore, better PFS and OS were observed when durvalumab was initiated within < 14 days after radiation [15].

Recently, the NCT03055715 trial of the “young DEGRO” working group of the DEGRO revealed a strong predictive value of the gross tumor volume (GTV) detected at the end of radiotherapy in the radiation boost re-planning CT in a multicenter retrospective study of 347 patients with locally advanced stage III NSCLC treated with radical concurrent chemoradiotherapy (*n* = 347 included patients, *n* = 177 with second CT scan for re-planning) [16].

In most cases, TNM staging derives from surgical interventions. However, initial tumor volume might be more important than T stage in patients treated with CRT without surgery. Such relations were also shown by other studies [17].

The primary objective of our multicenter study was to validate the predictive role of tumor volume change under CRT in stage III NSCLC as a predictor for a response to immunotherapy.

## Methods

### Study population, treatment, and participating institutions

This retrospective observational cohort study was conducted as a follow-up study of the NCT03055715 trial by the Young DEGRO Trial Group (yDEGRO). The former study included a total number of *n* = 347 patients who were treated with curative-intent radiation therapy (with/without chemotherapy) during the accrual period (January 1, 2010, to December 31, 2013) [16].

**Fig. 1:**
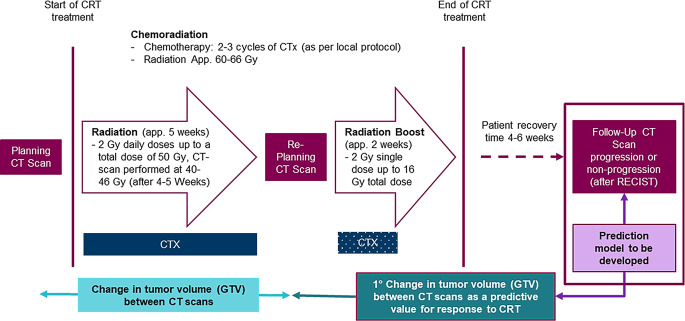
Overview of the data-generating process; *CTX* chemotherapy, *app.* application, *GTV* gross tumor volume, *CRT* chemoradiotherapy

Ten university centers participated in the present study (eight of these centers were part of the previous study). The inclusion period was 2015–2023. The data-generating process is depicted in Fig. 1. The treatment starts with the initial planning CT scan and enters the final stage after 40–50 Gy with a second re-planning CT scan intended to conclude the treatment with the radiation boost. The change in tumor volume is the supposed predictor of overall survival (OS). The GTV is defined as the volume of the primary tumor while lymph nodes are not considered.

Inclusion criteria were 1) an age of at least 18 years, 2) biopsy-proven locally advanced NSCLC (patients treated by primary CRT; stage III as determined by CT or PET scan), 3) at least two cycles of platinum-based chemotherapy concurrent with radiation therapy, 4) CT-based imaging of tumor volume during CRT, 5) a World Health Organization (WHO) performance status of 0–1, and 6) an estimated life expectancy of more than 12 weeks. Patients with prior exposure to any anti-PD‑1 or anti-PD-L1 antibody therapy; patients with an active or prior autoimmune disease or history of immunodeficiency; patients with evidence of severe or uncontrolled systemic diseases, including active bleeding, diatheses, or active infections including hepatitis B, C, and HIV; patients with evidence of uncontrolled illnesses such as symptomatic congestive heart failure, uncontrolled hypertension, or unstable angina pectoris; patients with any unresolved toxicity CTCAE > grade 2 from the prior CRT; and patients with active or prior documented inflammatory bowel disease (e.g. Crohn’s disease, ulcerative colitis) were excluded from the study.

The local ethics (reference number: 2016-122) and data protection committees of the participating institutions approved the study protocol and gave their positive vote, which was carried out in accordance with the declaration of Helsinki of 1975 (as revised in 2008).

Patient, treatment, and clinical data were extracted from the patients’ clinical records at the participating sites and collected using electronic case report forms (eCRF) which were stored in the RadPlanBio database of the German Cancer Consortium (DKTK) and the German Cancer Research Center (DKFZ) [18, 19]. The platform provides a basis to collect data in radiation oncology and is hosted at the University Hospital Carl Gustav Carus, TU Dresden University of Technology. An intensive validation process was performed to check for implausible and incorrect values. This process was based on statistical approaches and spot checks. Written informed consent of all patients was available for data acquisition and analysis. Staging was based on the TNM classification of malignant tumors (8th edition 2016).

### Detection and definition of gross tumor volume and toxicity

The GTV included the gross tumor volume (without lymph nodes) as detectable in the planning CT or FDG-PET/CT and was reported in milliliters. Where FDG-PET was available, FDG-PET-CT co-registration was hardware (integrated PET-CT scanner) or software based (with the need for repositioning of the patient), according to the equipment situation of the institutions. The baseline GTV1 was delineated in the planning CT, which was obtained before the start of RT and correlated with PET, if existing. GTV2 was obtained from the re-planning CT after the patients had received 40–50 Gy. The assessment of tumor response after chemoradiotherapy was based on the first routine follow-up scan using the RECIST 1.1 criteria.

In survival plots, we defined low and high values according to the 25% and 75% quantile.

### Statistics

To account for heterogeneity in GTV contouring, we used frailty survival methods with study center as the shared frailty. This method includes a random term to account for systematic institutional variation in delineation.

Absolute and relative GTV changes before/during CRT were correlated with OS to predict the response to CRT with sequential immunotherapy. This association was assessed by computing hazard ratios (HR) from Cox regression models using the coxph() function of the R survival package. Here, an HR of 0.96 of the absolute volume change can be interpreted as a reduction in the risk of experiencing an event of 4% per milliliter tumor shrinkage. If the tumor shrinks by more than 1 ml, the corresponding risk reduction is higher.

All other analyses are secondary.

Our models were additionally adjusted for UICC stage, chemotherapy, age, RT dose (given by treatment), histology (adenocarcinoma or squamous cell carcinoma), grading, and pulmonary comorbidities.

For illustrative purposes, we plotted Kaplan–Meier curves. The 25%, 50%, and 75% quantiles were used to define the patient groups with low, intermediate, and high GTV values as well as strong, intermediate, and weak GTV decreases from GTV1 before RT and GTV2 during RT. For survival comparisons, HR values are reported with a 95% confidence interval (CI).

Deviations from the assumption of random residuals were assessed visually by plotting the Martingale residuals. In addition, we computed the linear metric association of tumor volume and its alteration during treatment by applying linear regression models.

Further, the statistical analyses also include models considering only complete observations without missing data.

A significance level of 5% was used. All statistical analyses were performed using R 4.1.2 (R Core Team 2021).

## Results

### Patient characteristics

The dataset consists of 203 patients characterized by various demographic and clinical attributes. After excluding implausible values, 189 patients remained for analysis, as detailed in Table 1. The gender distribution was 65% male (123 patients) and 35% female (66 patients). In the durvalumab subgroup (*n* = 86), the distribution was similar, with 62% male and 38% female. The median age at diagnosis was 65.5 years overall and 64 years for the durvalumab group, with an interquartile range (IQR) of 59–71 years for both.

Regarding tobacco use, 18% were non-smokers, 36% were smokers, and 32% were ex-smokers; 13% had unknown smoking status. The durvalumab group had similar smoking proportions.

**Table 1:** Patient characteristics

		All patients	Durvalumab-treated patients	Patients without durvalumab treatment
GTV1 (ml)	1st quartile	61.36	50.75	75.59
	Median	102.79	88.70	143.00
	Mean	145.29	14.90	162.11
	3rd quartile	204.93	175.21	216.54
GTV2 (ml)	1st quartile	32.93	26.59	38.70
	Median	70.45	55.50	83.40
	Mean	99.58	82.82	113.40
	3rd quartile	126.85	106.83	153.10
Absolute volume change (ml)	1st quartile	5.598	4.00	6.45
	Median	25.715	24.79	26.48
	Mean	45.701	42.08	48.69
	3rd quartile	66.347	59.80	78.96
Radiation dose (Gy)	1st quartile	60.00	62.25	60.00
	Median	66.00	66.00	66.00
	Mean	63.75	64.60	63.03
	3rd quartile	66.00	66.60	66.00
Radiation technique (n)	VMAT	120	51	69
	IMRT	59	30	29
	Other	8	5	3
Chemotherapy (n)	Cisplatin	105	53	52
	Vinorelbine	111	55	56
	Paclitaxel	54	11	43
	Etoposide	11	10	1
	Carboplatin	84	31	53
Histology (n)	Squamous cell carcinoma	105	44	61
	Adenocarcinoma	76	37	39
	Unknown	8	5	3
Grading (n)	G1	2	0	2
	G2	64	33	31
	G3	62	36	26
	G4	2	1	1
	Unknown	59	16	43
PD-L1 (n)	< 1%	38	4	34
	≥ 1%	103	77	26
	Unknown	48	5	43
UICC (n)	III	6	0	6
	IIIA	70	32	38
	IIIB	92	42	50
	IIIC	21	12	9
T stage (n)	T1	11	6	5
	T2	10	5	5
	T2a	7	2	5
	T2b	10	3	7
	T3	44	25	19
	T4	104	44	60
N stage (n)	Nx	6	2	4
	N0	27	12	15
	N+	3	1	2
	N1	21	11	10
	N2	77	35	42
	N3	54	24	30
PET-based planning (n)	No	9	3	6
	Yes	180	83	97
Age at onset (years)	1st quartile	59.00	69.00	59.00
	Median	65.50	64.00	66.50
	Mean	65.07	64.67	65.41
	3rd quartile	71.25	71.00	72.00
Pulmonary comorbidities (n)	Yes	82	49	33
	No	106	37	69
	Unknown	1	0	1
Gender (n)	Female	66	33	33
	Male	123	53	70
Smoking (n)	Never	35	18	17
	Current	69	28	41
	Past	60	34	26
	Unknown	25	6	19
Durvalumab (n)	Yes	86	86	0
	No	79	0	79
	Unknown	24	0	24

*GTV1* pre-treatment gross tumor volume, *GTV2* replanning gross tumor volume, *VMAT* volume modulated arc therapy, *IMRT* intensity-modulated radiation therapy

Cancer staging according to the Union for International Cancer Control (UICC) showed 49% in stage IIIB, 37% in stage IIIA, and 11% in stage IIIC, with similar distributions in both groups. Squamous cell carcinoma (SCC) was the most common histological subtype, present in 55% of all patients and in 51% of those treated with durvalumab. Adenocarcinoma (AC) was diagnosed in 40% of patients. The histological subtype was unknown for 5% overall and for 6% in the durvalumab subgroup.

Regarding PD-L1 status, 20% had less than 1% expression, 54% had 1% or greater, and 25% were unknown. In the durvalumab group, 90% had PD-L1 expression of ≥ 1%. Four patients (5%) with PD-L1 levels below 1% were treated with durvalumab.

Overall, 86 patients (45%) were treated with durvalumab, while 79 patients (42%) did not receive durvalumab therapy. The majority of tumors were classified as grade G2 (34% or 64 patients) and grade G3 (33% or 62 patients), with similar proportions in the durvalumab-treated group. Grades G1 and G4 were less common, each accounting for 1% of the patients (2 patients each). Tumor grading was unknown for a substantial portion (59 patients; 31%).

Regarding T status, T4 stage tumors were most prevalent, found in 104 patients (55%) treated with or without durvalumab and in 44 patients (51%) receiving durvalumab. Other T stages ranged widely from T1 to T3. The most common N stage was N2 with 77 patients (41%), followed by N3 with 54 patients (29%). An N0 status was observed in 27 patients (14%) and N1 in 21 patients (11%).

Pulmonary comorbidities were present in 82 patients (43%) considering all included cases and in 49 patients (56%) in the durvalumab-treated group, with slightly fewer patients without pulmonary comorbidities in this group.

Radiation planning was FDG-PET/CT based in the majority of cases for both treatment groups (all patients 95%; durvalumab group 96%). The most frequently used radiation technique was VMAT (all patients: *n* = 120, 63%; durvalumab group: *n* = 51, 59%), followed by IMRT. Regarding radiation dose, the median dose was 66 Gy, with 25th and 75th percentiles of 60 Gy and 66 Gy considering all patients (durvalumab group: median dose 66 Gy; 25th and 75th percentiles 62.25 Gy and 66.60 Gy, respectively).

Chemotherapy regimens showed that cisplatin was the most commonly used chemotherapeutic drug in combination with vinorelbine (all patients: *n* = 111, 60%; durvalumab group: *n* = 55, 64%). Paclitaxel, etoposide, and carboplatin were also used less frequently.

### Intra-therapeutic GTV changes and the association with overall survival

The median OS for the entire cohort was 13.7 months (Table 2). At the time of analysis, 52 patients had died and 82 patients had experienced a progression.

**Table 2:** Median overall survival (months)

		All patients	Durvalumab treated patients
GTV1	Low	19.77	17.80
	Intermediate	12.95	17.52
	High	13.37	15.60
GTV2	Low	14.37	17.80
	Intermediate	13.42	17.52
	High	14.47	15.60
Absolute volume change	Low	14.47	21.57
	Intermediate	13.78	14.83
	High	13.00	15.90
Total		13.70	15.75

The mean GTV1 before RT was 145.29 ml, with 25th, 50th, and 75th percentiles of 61.36 ml, 145.29 ml, and 204.93 ml, respectively. Before initiation of the radiation boost, the mean GTV2 was 99.58 ml, with the 25th, 50th, and 75th percentiles at 32.93 ml, 70.45 ml, and 126.85 ml, respectively.

**Fig. 2:**
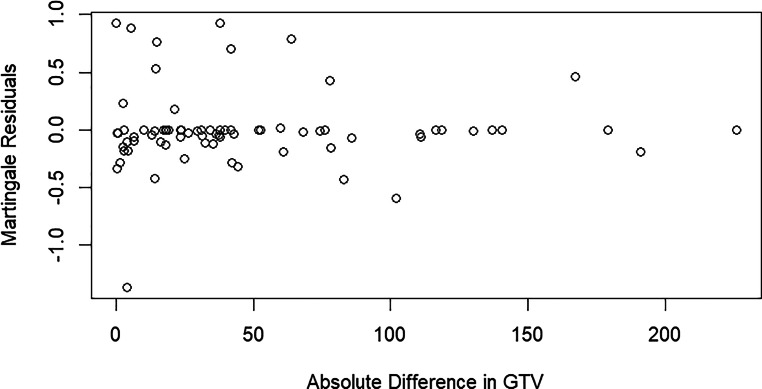
Martingale residual plot of the absolute differences between GTV1 and GTV2

The mean absolute difference between the two volumes was 45.70 ml, with the 25th, 50th, and 75th percentiles at 5.59 ml, 25.71 ml, and 66.34 ml, respectively, for all patients and 42.08 ml with an IQR of 4.00–59.8 ml for patients treated with durvalumab after CRT. Figure 2 shows a scatter plot of the relationship between the absolute difference in GTV1 and GTV2 and Martingale residuals. The Cox proportional hazards model generally fits the data well for most patients, as indicated by the concentration of residuals around 0. Figures S1 und S2 show the Kaplan–Maier curves of high, intermediate, and low absolute GTV1 and GTV2 volumes. Figure S3 illustrates the Kaplan–Meier plot of high, intermediate, and low absolute changes between the two volumes.

Median OS was 19.77 months, 12.95 months, and 13.37 months for patients with a low, intermediate, and high baseline GTV1 before RT, respectively. Moreover, the median OS was 14.47 months, 13.42 months, and 14.37 months for patients with a high, intermediate, and low GTV2, respectively, before the initiation of boost RT. The respective HR values can be found in Tables 3 and 4. For patients treated with durvalumab, the pretreatment GTV had no significant association with OS (HR = 1.01, 95% CI 0.97–1.04; *p* = 0.46). In contrast, the absolute volume change between GTV1 and GTV2 was significantly correlated with OS, with an HR of 0.96 per millimeter (95% CI 0.91–1.04; *p* = 0.03) for patients treated with durvalumab. To adjust for a potential immortal time bias, we set 42 days as the landmark based on the latest administration of durvalumab in the PACIFIC trial [15]; the resulting HR was 0.96 (0.92–0.99; *p* = 0.031).

**Table 3:** Hazard ratios for all patients with the outcome of overall survival

		Hazard ratio	95% confidence interval	*p*-value
GTV1	–	0.999	0.996–1.002	0.49
Absolute volume change	–	1.004	0.997–1.011	0.26
Radiation dose	–	0.954	0.878–1.037	0.27
Histology	Squamous cell carcinoma	1 (reference)	–	–
	Adenocarcinoma	0.675	0.339–1.344	0.26
Grading	G1/2	1 (reference)	–	–
	G3/4	1.210	0.573–2.556	0.62
UICC	IIIA	1 (reference)	–	–
	IIIB/C	1.639	0.812–3.305	0.17
Age at onset	–	0.992	0.956–1.030	0.69
Pulmonary comorbidities	Yes	0.927	0.468–1.838	0.83
	No	1 (reference)	–	–
Smoking	Never	1 (reference)	–	–
	Current	1.118	0.338–3.705	0.85
	Past	2.180	0.649–7.323	0.21
Durvalumab	Yes	0.454	0.209–0.990	0.047
	No	1 (reference)	–	–

**Table 4:** Hazard ratios for durvalumab treated patients with the outcome of overall survival

		Hazard ratio	95% confidence interval	*p*-value
GTV1	–	1.013	0.979–1.048	0.46
Absolute volume change	–	0.955	0.916–0.996	0.03
Radiation dose	–	0.969	0.744–1.263	0.82
Histology	Squamous cell carcinoma	1 (reference)	–	–
	Adenocarcinoma	0.659	0.098–4.429	0.67
Grading	G1/2	1 (reference)	–	–
	G3/4	0.870	0.094–8.100	0.90
UICC	IIIA	1 (reference)	–	–
	IIIB/C	1.220	0.217–6.854	0.17
Age at onset	–	0.935	0.828–1.055	0.27
Pulmonary comorbidities	Yes	1.193	0.144–9.853	0.87
	No	1 (reference)	–	–
Smoking	Never	1 (reference)	–	–
	Current	0.027	0.0003–2.497	0.12
	Past	0.341	0.004–28.718	0.63

**Fig. 3:**
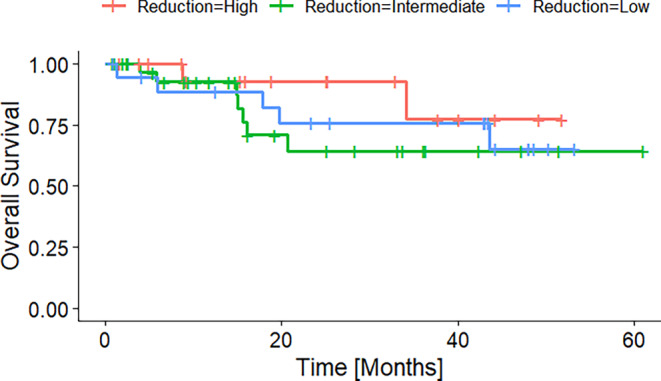
Kaplan–Meier plot of OS according to absolute GTV difference quantiles before radiotherapy of the durvalumab subgroup. High, intermediate, and low GTV differences referring to the 25% and 75% quantiles

**Fig. 4:**
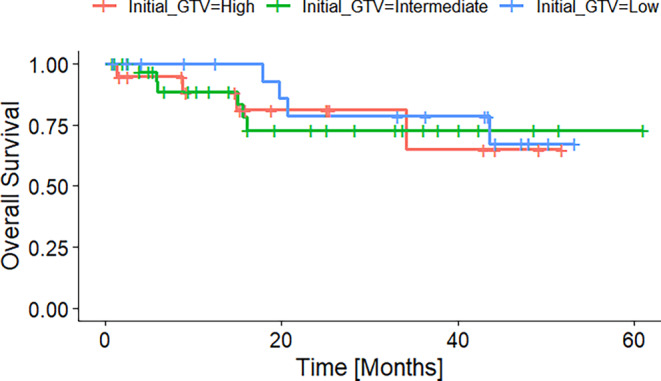
Kaplan–Meier plot of OS according to absolute GTV1 before radiotherapy of the durvalumab subgroup. High, intermediate, and low GTV1 referring to the 25% and 75% quantiles

**Fig. 5:**
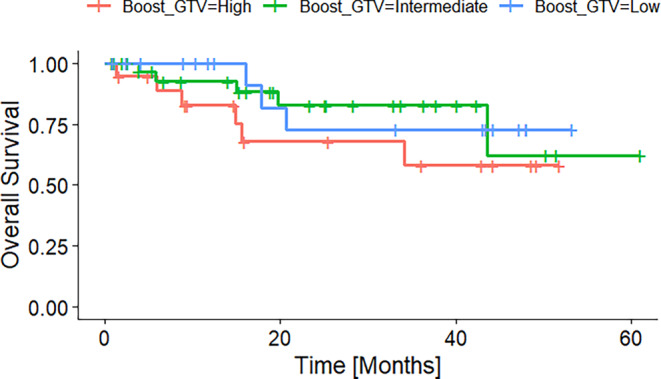
Kaplan–Meier plot of OS according to absolute GTV2 before radiotherapy boost of the durvalumab subgroup. High, intermediate, and low GTV2 referring to the 25% and 75% quantiles

**Fig. 6:**
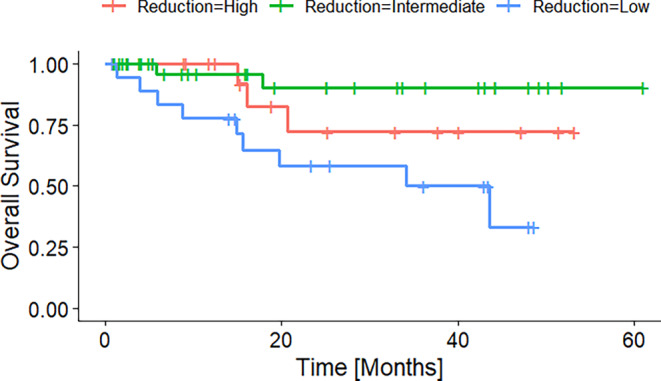
Kaplan–Meier plot of OS according to relative GTV change during radiotherapy (from GTV1 to GTV 2) of the durvalumab subgroup. Low, intermediate, and high GTV decrease referring to the 25 and 75% quantiles

Figure 3 shows the overall survival of patients grouped into patients with a high, intermediate, and low absolute tumor volume reduction. The groups were formed according to the first and third quantile. Figures 4 and 5 display the Kaplan–Meier curves for high, intermediate, and low absolute volumes of GTV1 and GTV2 of the durvalumab subgroup. Figure 6 presents the Kaplan–Meier plot for the absolute changes between the two volumes, categorized as high, intermediate, and low for patients treated with durvalumab.

The HRs regarding administered radiation dose, histology, and grading can be found in Tables 3 and 4, respectively.

In short, UICC stage, age at onset, pulmonary comorbidities, and smoking status were not found to be prognostic factors in terms of survival. Only durvalumab treatment was significantly associated with improved OS, with an HR of 0.454 (95% CI 0.209–0.990; *p* = 0.047). All results of our Cox regression analysis can be found in Tables 3 and 4.

We performed an additional patterns-of-failure analysis to examine the distribution of recurrences in all patients and specifically in those treated with durvalumab. Among all patients, 25 experienced local recurrence, 3 had regional recurrence, and 29 developed distant metastases (Table 5). In the durvalumab-treated group, 9 local recurrences, 1 regional recurrence, and 17 distant metastases were observed (Fig. 6). To illustrate the relationship between intrathoracic progression and distant progression with OS, we computed Kaplan–Maier curves. These Kaplan–Maier curves can be found in the supplement section (figures S4 and S5).

**Table 5:** Sites of treatment failure

	All patients	Durvalumab-treated patients
Local recurrence	25	9
Regional recurrence	3	1
Distant metastasis	29	17

## Discussion

In our analysis there was no evidence of an impact of the pre-treatment GTV on OS in either treatment group. These findings are consistent with the results of the study by Ball et al., who, after adjusting for the T and N stages, also did not find a significant association between pre-treatment GTV and survival after radical RT [20].

In a study by Kanzaki et al., a significant impact of the pre-treatment GTV on OS was reported after RT for a continuous increase of 10 cm^3^ [21]. Our own data indicate an association of absolute volume changes on survival but reach statistical significance only in the durvalumab subgroup. In accordance with these findings, durvalumab treatment was associated with improved OS.

To the authors’ knowledge, the TORCH study, which includes a total of *n* = 189 patients with inoperable stage III NSCLC treated by definitive CRT from ten German radiation oncology university centers is one of the largest multicenter retrospective analyses of the prognostic impact of pre-treatment GTV, GTV during RT, and GTV changes for a response to immunotherapy during the course of therapy. The use of real-life data in our study enables us to assess the value of tumor volume change in an unbiased everyday setting.

Comparable studies often include only a small number of patients, and data quality is limited due to heterogeneous GTV definition and the methodology of tumor volume detection.

Moreover, most studies include patients whose GTVs were determined after neoadjuvant chemotherapy. In three studies, the tumor volumes of the primary tumor were combined with the affected lymph nodes [22–24]. In contrast, only patients without previous chemo- or surgical therapy who were treated with definitive RCT in curative intent were included in our study.

In our data, patients treated with durvalumab with high pre-RT GTVs had inferior median OS rates compared to patients with intermediate and low pre-RT GTVs. These results correspond to other studies evaluating the pre-treatment GTV and its impact on outcome after RT such as analyses form Martel, Kim, and Bradley et al., who reported a strong influence of the baseline GTV on OS and tumor control [17]. Basaki et al. also identified primary and total tumor volumes as prognostic factors for survival. The nodal tumor volume did not significantly affect survival in their study [25].

Treatment with durvalumab became the standard of care after the PACIFIC trial had demonstrated that PFS and OS in patients with UICC stage III non-small cell lung cancer (NSCLC) can be improved with consolidative immunotherapy following definitive concurrent chemoradiotherapy (CRT) [15]. Our results further suggest that the absolute GTV change could be a predictor in terms of survival for patients treated with durvalumab.

Further, Kanzaki et al. found baseline GTV before RT to be an independent prognostic factor in patients with adenocarcinoma [21], which indicates a potentially important role of histology in the GTV-guided survival prediction. Due to the fact that the association between histology and GTV has not been widely evaluated in the literature, the impact of histology might be underestimated. After adjusting for different parameters including histology, our statistical analyses did not reveal the histological subtype to be a significant factor, in line with the findings of the prior young DEGRO study [16]. When the impact of histological subtypes on OS was analyzed in our data, AC had a better survival probability compared to SCC patients without reaching statistical significance.

While the summarized evidence favors the prognostic value of the pre-treatment GTV overall, the predictive value of the GTV detected during RT is still unclear [17, 26, 27]. There are only a few studies with a limited number of evaluated patients investigating the prognostic impact of the intra-therapeutic GTV. In general, no final agreements can be found in the literature concerning intra-therapeutic volume changes [16].

We re-evaluated GTV changes during RT between 40 and 50 Gy. By contrast, the GTV measurement timepoints in the literature vary between 2 weeks after the start of RT and 4 weeks after the end of RT [17]. Therefore, re-evaluating the GTV during RT between 40 and 50 Gy to adapt the treatment plan for tumor volume changes appears feasible. Significant tumor volume changes after 30–50 Gy were also mentioned in previous studies [28, 29].

The prediction of outcomes based on GTV changes remains indecisive in the literature [17]. While some studies [21, 27, 29] showed an association between tumor volume reduction and improved OS or PFS, others [30] could not validate these findings. Furthermore, there are conflicting results indicating inferior survival in patients with a higher tumor volume reduction during RT [30].

The large number of included stage III NSCLC patients (*n* = 189) exceeds the patient number of most of the published studies. Nevertheless, despite the fact that inclusion and patient selection criteria in this study were comprehensively selected, selection effects and bias at the individual center level must be taken into account.

Although the patient cohort in this study was relatively homogenous in terms of treatment, with a majority of 87% of patients having received more than 60 Gy and conventional fractionation in 96% with simultaneous CRT in 100% of patient cases, a certain heterogeneity level regarding radiation dose, fractionation dose, and CT timing needs to be considered. Additionally, the initiation of immunotherapy after CRT might also be heterogeneous between the study centers due to differing treatment workflows.

Furthermore, the retrospective design of this study limits its explanatory power compared to a prospective study. Our study focused on a cohort of UICC stage III patients in an unequal distribution. Moreover, potential variability could be caused by inter-center inconsistencies and treatment regimen variability, and differing imaging methods for GTV re-evaluation could contribute to noisy data. This was addressed by including the study center as the frailty. Furthermore, the initial size of the tumor differed between patients with and without durvalumab treatment. This was addressed by including the initial GTV1 as a covariate in the Cox regression model. GTV definition and 3D radiation planning in this study were uniform, and imaging for GTV1 and GTV2 detection was performed similarly at the centers. However, potential variations in FDG-PET/CT co-registration methods need to be taken into consideration. FDG uptake was evaluated during thoracic RT to determine whether significant changes in FDG uptake or tumor volume could be measured early enough to adapt the RT plan. During RT, PET/CTs were performed every 7 fractions. An average 50% decrease in maximal standard uptake value (SUVmax) was observed around 40–45 Gy (i.e., during week 5 of RT) [31], which aligns with the timing of the replanning CT scans performed by the study centers.

Nevertheless, a certain level of uncertainty and difficulty caused by the definition of the GTV excluding lymph nodes remained, leading to a difficult delineation process for the trialists. Especially in stage III NSCLC, the separation between the GTV and the adjunct lymph nodes can be challenging, even for experienced radiation oncologists [32].

In our study we did not measure volume changes in lymph nodes which might also respond to radiotherapy. However, the primary tumor volume is a more suitable surrogate, as false classifications due to inflammatory nodes are less likely to bias our results. In addition, lymph nodes contribute only minorly to the total tumor volume, even more as they are encapsulated. In our cohort, only three patients experienced a progression in lymph nodes, which underscores their subordinate role compared to the primary tumor when it comes to the estimation of total tumor burden.

The prospective phase II RTEP7-IFCT-1402 study investigated local tumor control rates for patients treated with adaptive RT (up to 74 Gy) or standard RT (66 Gy) according to FDG-PET residual tumor uptake at 42 Gy. In their findings, the PET-guided RT boost improved local control without increased severe adverse events compared to standard RCT. Additionally, both groups underwent induction chemotherapy followed by concurrent RCT with platinum-based regimens [33]. In our retrospective study, none of the patients were treated with induction chemotherapy. Furthermore, FDG-PET/CT was used for initial treatment planning and not for re-planning. Based on the findings of Vera et al. [33], future studies could focus on the predictive value of tumor volume shrinkage after induction chemotherapy in combination with concurrent CRT.

In our study, the absolute tumor volume reduction had a predictive value in terms of survival for durvalumab-treated patients. To our knowledge, there are only a few studies dealing with the predictive role of GTV changes for the survival outcome of patients treated with durvalumab. This could be explained by the fact that most studies on this topic were conducted in the period before the era of immunotherapy [17]. A current monocenter study conducted by Lee et al. investigated the prognostic value of the pretreatment and re-planning GTV of 227 stage III NSCLC patients who underwent CRT followed by durvalumab. In their study, relative GTV regression was found to be a promising predictor of survival [34]. Future research is needed to investigate the prognostic role of GTV changes in durvalumab patients.

## Conclusion

High absolute GTV shrinkage was associated with improved median OS in our patient cohort treated with durvalumab. We did not observe an association between absolute volume change and OS during RT in the overall cohort. This finding did not completely confirm our initial hypothesis; on the contrary, it gives hope to patients without a good tumor response during the first phase of RT.

Our study also highlighted that histological subtype, grading, UICC stage, age at onset, pulmonary comorbidities, and smoking status did not significantly impact survival, contrasting with the significance of durvalumab treatment. Our findings support the notion that tumor volume reduction during RT can be a promising predictor of survival, especially since consolidative treatment with durvalumab is the current standard of care in the treatment of locally advanced NSCLC.

Future research is needed to further investigate the prognostic role of GTV changes, particularly in the era of immune checkpoint inhibitor treatment, to optimize therapeutic strategies for patients with stage III unresectable NSCLC.

## Supplementary Information

The supplementary figures provide additional insights into the impact of tumor volume on survival outcomes. Figure S1presents a Kaplan–Meier plot of overall survival (OS) based on absolute GTV1 before radiotherapy, categorized into high, intermediate, and low groups according to the 25th and 75th percentiles. Similarly, Figure S2 illustrates OS based on absolute GTV2 before the radiotherapy boost, following the same categorization. Figure S3 shows OS in relation to relative GTV changes during radiotherapy (from GTV1 to GTV2), with low, intermediate, and high GTV decreases defined by the 25th and 75th percentiles. Figure S4 depicts intrathoracic progression-free survival (PFS) in patients treated with durvalumab, stratified according to absolute tumor volume reduction. Lastly, Figure S5 presents distant metastasis-free survival (DMFS) in durvalumab-treated patients, also stratified by absolute tumor volume reduction.

## Abbreviations

AC: Adenocarcinoma
CHT: Chemotherapy
CI: Confidence interval
CRF: Case report form
CRT: Chemoradiotherapy
CT: Computer tomography
DEGRO: German Society of Radiation Oncology
DKFZ: German Cancer Research Center
DKTK: German Cancer Consortium
ECOG: Eastern Cooperative Oncology Group
FDG-PET/CT: Fluorodeoxyglucose positron-emission tomography
GTV: Gross tumor volume
HR: Hazard ratio
IMRT: Intensity-modulated radiation therapy
NSCLC: Non-small cell lung cancer
OS: Overall survival
PD-L1: Programmed death-ligand 1
RECIST: Response Evaluation Criteria in Solid Tumors
RKI: Robert Koch Institute
RT: Radiation therapy
SCC: Small cell carcinoma
SCLC: Small cell lung cancer
SD: Standard deviation
SIB: Simultaneous integrated boost
TNM: Tumor node metastasis
UICC: Union of International Cancer Control
VMAT: Volume modulated arc therapy
