# Supplementary material for: Tumor volume change at radiation boost planning to estimate the response to chemoradiotherapy in stage III unresectable NSCLC (TORCH): a multicenter retrospective observational study
Source: Strahlenther Onkol. 2025 Mar 3;201(10):1001–13. doi: 10.1007/s00066-025-02374-3 (PMC12488809; doi:10.1007/s00066-025-02374-3)
Supplement: Supplementary file 1 — The supplementary figures provide additional insights into the impact of tumor volume on survival outcomes. Figure S1presents a Kaplan–Meier plot of overall survival (OS) based on absolute GTV1 before radiotherapy, categorized into high, intermediate, and low groups according to the 25th and 75th percentiles. Similarly, Figure S2 illustrates OS based on absolute GTV2 before the radiotherapy boost, following the same categorization. Figure S3 shows OS in relation to relative GTV changes during radiotherapy (from GTV1 to GTV2), with low, intermediate, and high GTV decreases defined by the 25th and 75th percentiles. Figure S4 depicts intrathoracic progression-free survival (PFS) in patients treated with durvalumab, stratified according to absolute tumor volume reduction. Lastly, Figure S5 presents distant metastasis-free survival (DMFS) in durvalumab-treated patients, also stratified by absolute tumor volume reduction. [file 66_2025_2374_MOESM1_ESM.docx]

# Supplement

**
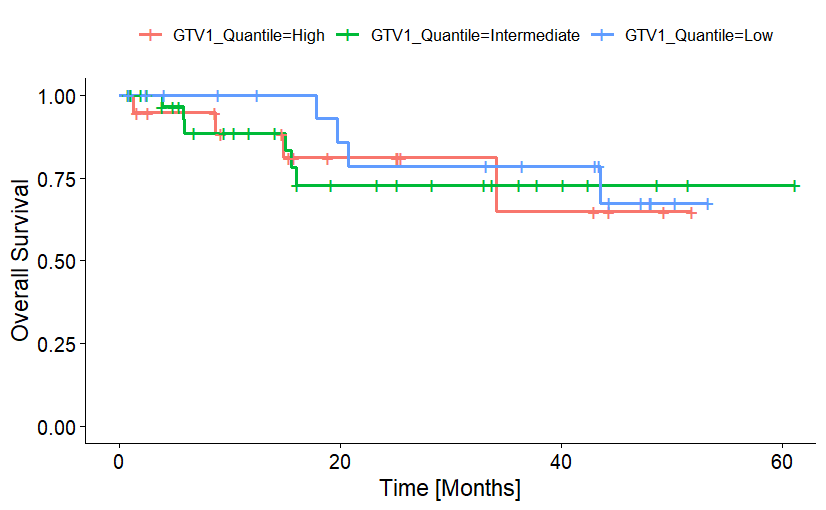
**

**Figure S1:** Kaplan–Meier plot of OS according to absolute GTV1 before radiotherapy. High, intermediate, and low GTV1 referring to the 25 and 75% quantiles.

**
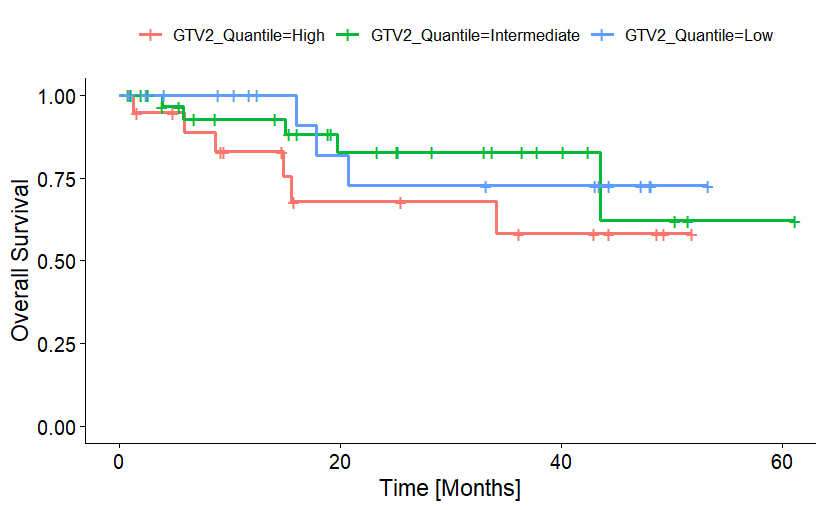
**

**Figure S2:** Kaplan–Meier plot of OS according to absolute GTV2 before radiotherapy boost. High, intermediate, and low GTV2 referring to the 25 and 75% quantiles.


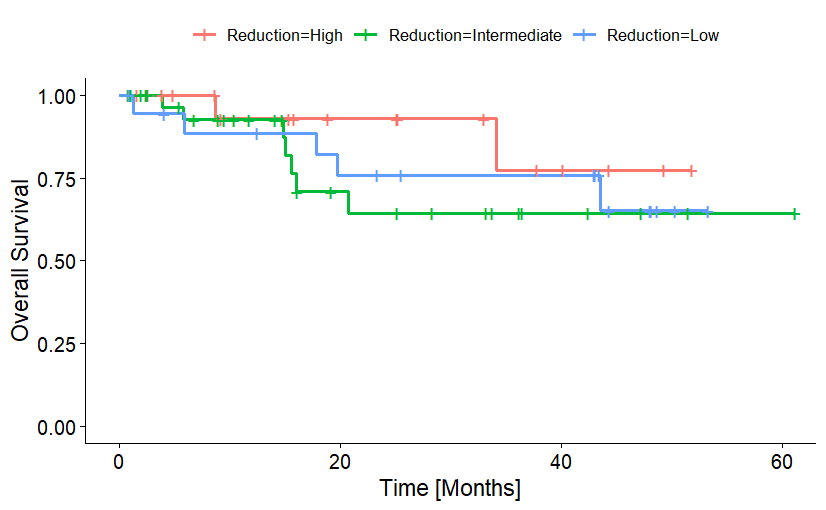


**S3:** Kaplan–Meier plot of OS according to relative GTV change during radiotherapy (from GTV1 to GTV2). Low, intermediate, and high GTV decrease referring to the 25 and 75% quantiles.


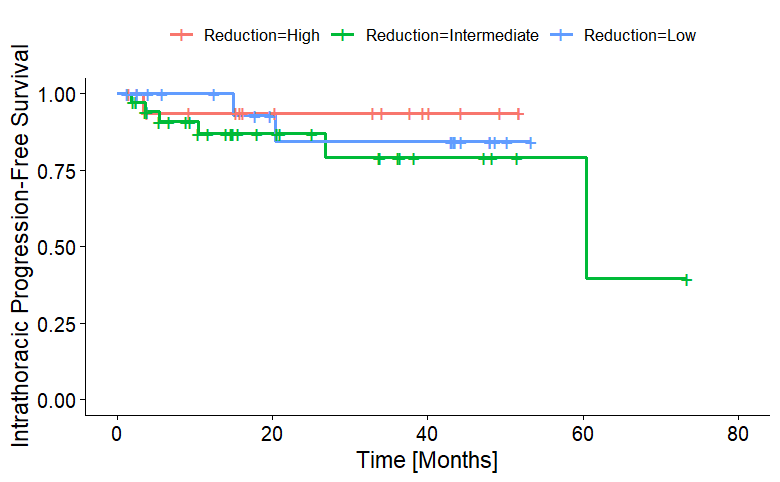


**S4:** Intrathoracic Progression-Free Survival of durvalumab treated patients, stratified according to absolute tumor volume reduction.


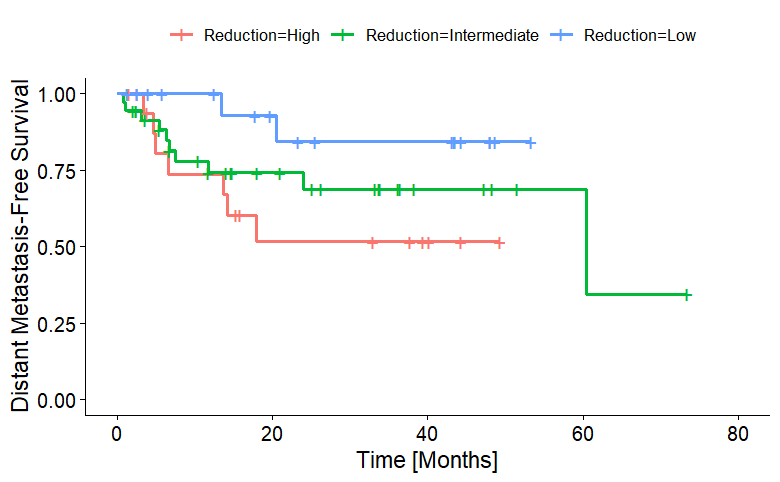


**S5:** Distant Metastasis-Free Survival for Patients treated with durvalumab, stratified according to absolute tumor volume reduction.
